# Hydrogels—A Promising Materials for 3D Printing Technology

**DOI:** 10.3390/gels9030260

**Published:** 2023-03-22

**Authors:** Gobi Saravanan Kaliaraj, Dilip Kumar Shanmugam, Arish Dasan, Kamalan Kirubaharan Amirtharaj Mosas

**Affiliations:** 1 Centre for Nanoscience and Nanotechnology, Sathyabama Institute of Science and Technology, Chennai 600 119, India; 2 FunGlass—Centre for Functional and Surface Functionalised Glass, Alexander Dubcek University of Trencin, 91150 Trencin, Slovakia

**Keywords:** polymer hydrogels, wound dressing, regenerative medicine, pH-sensitive hydrogel, thermo-sensitive hydrogel

## Abstract

Hydrogels are a promising material for a variety of applications after appropriate functional and structural design, which alters the physicochemical properties and cell signaling pathways of the hydrogels. Over the past few decades, considerable scientific research has made breakthroughs in a variety of applications such as pharmaceuticals, biotechnology, agriculture, biosensors, bioseparation, defense, and cosmetics. In the present review, different classifications of hydrogels and their limitations have been discussed. In addition, techniques involved in improving the physical, mechanical, and biological properties of hydrogels by admixing various organic and inorganic materials are explored. Future 3D printing technology will substantially advance the ability to pattern molecules, cells, and organs. With significant potential for producing living tissue structures or organs, hydrogels can successfully print mammalian cells and retain their functionalities. Furthermore, recent advances in functional hydrogels such as photo- and pH-responsive hydrogels and drug-delivery hydrogels are discussed in detail for biomedical applications.

## 1. Introduction

**Figure 1:**
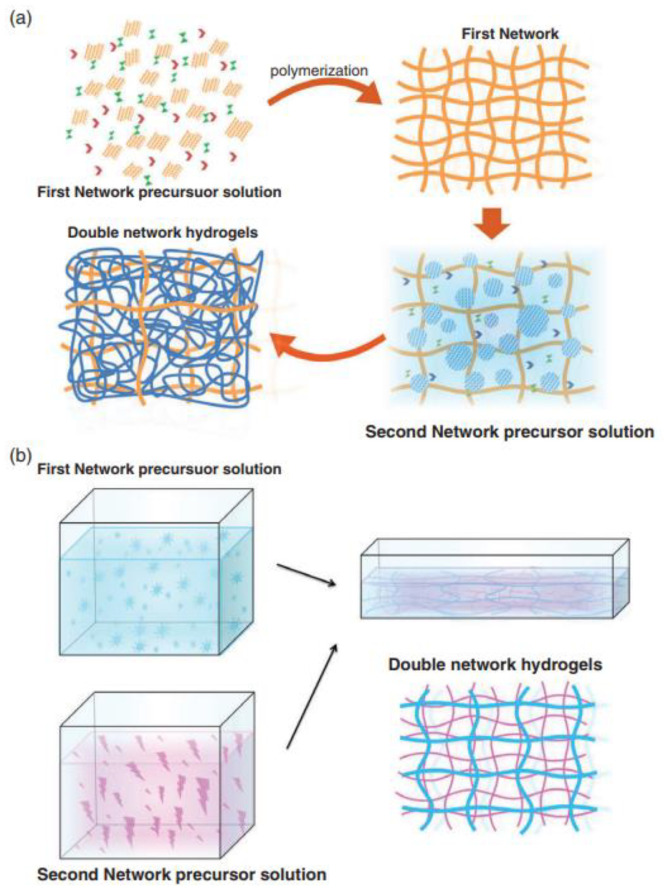
Schematic view of double-network hydrogel preparation by chemical-chemical crosslinking. (**a**) two-step polymerization method and (**b**) molecular stent process [4]. Copyright received.

Hydrogels are three-dimensional (3D) network polymers that can swell and store a significant amount of water to maintain their structure due to the physical and chemical cross-linking nature of the polymer chains. The name “hydrogel” was first coined by Wichterle and Lim in 1960 [1]. The ideal characteristic of a hydrogel is to hold water representing at least 10% of the total weight of the material. Hydrogel possesses a degree of flexibility that resembles actual tissue owing to its significant water content. In general, hydrogels are highly hydrophilic in nature due to the presence of -COOH, -NH_2_, -OH, -CONH, -CONH_2_, and -SO_3_H functional groups. They undergo a severe volume phase transition based on the chemical and physical stimuli response. Suitable chemical or biochemical stimuli include dissolved ions, pH, and chemical composition changes. Examples of physical stimuli include changes in the magnetic field, electric field, pressure, temperature, and light intensity. In particular, most of the changes in the hydrogels are reversible, and therefore, the hydrogels tend to return to their initial state after the stimulus is removed. Chen et al. prepared double-network hydrogels through a two-step polymerization process. The first step involves the formation of covalently crosslinked networks (Figure 1). Then, the first polymerized network is immersed in a second precursor solution containing second-network monomers, crosslinkers, and initiators [2]. Upon the swelling process, the second step network reactant diffuses into the first hydrogel polymerization network to create a loosely crosslinked network. In addition, Nakajima et al. fabricated a tough double-layer network hydrogel with two neutral polymers by a molecular stent method [3].

Various natural and synthetic polymers have been reported as hydrogel-based sensors with chemical molecular chains linked by covalent bonds as well as physical molecular chains linked through noncovalent bond interactions to obtain 3D network structures [5]. These synthetic and natural polymers can be either used as base materials to enhance the mechanical properties of the material or to explore its unique physical, chemical, and biological properties in various applications including chemical detection, stimuli responsiveness, self-healing, and conductivity. Various polymers have been utilized effectively as hydrogel-based sensors including polyacrylamide (PAA), polyvinyl imidazole (PVIm), polyethylene glycol (PEG), polyvinyl alcohol (PVA), and polyvinylpyrrolidone (PVP) [6].

These natural and synthetic polymers are widely used in many fields, including biotechnology, biomedical, separation science, and the pharmaceutical industry. In particular, such materials perform functions relevant to a wide range of biological interests and are currently used for manufacturing tissue engineering scaffolds, stretchable devices, contact lenses, drug delivery systems, hygiene products, and wound dressings. However, such applications strongly rely on the characteristic properties of the respective hydrogels. For instance, thermal, pH, and electrosensitive hydrogels have received a significant amount of attention in drug delivery applications. Furthermore, hydrogels are used effectively in heavy metal removal and smart packaging systems. However, because of their limited fabrication approaches to realizing end-use products, further potential applications remain elusive. In recent years, significant research attention has been given to utilizing hydrogels as feedstock materials in modern emerging technologies such as additive manufacturing (AM) or 3D printing. The advent of such technology has enabled new possibilities to manufacture geometrically highly complex structures directly from computer inputs, i.e., digital models [7]. Nevertheless, considering the geometric complexity and resolution of printed parts using hydrogels as feedstock is still an open issue and needs to be further investigated. Hence, the present review provides an overview of the range of hydrogels and a summary of various 3D printing techniques with hydrogels as printing materials. Moreover, current limitations, potential applications, and future perspectives are also provided.

## 2. Classification of Hydrogels

Hydrogels can be classified into several groups based on their structure, and properties including chemical (pH, glucose, oxidant) physical (temperature, pressure, light), and biological (antigen, enzyme, ligand) responses.

○ Natural hydrogels
○ Synthetic polymers
○ Functional hydrogels
○ Nanocomposite hydrogels
○ Conductive hydrogels
○ Photoresponsive hydrogels
○ Synthetic hydrogels

### 2.1. Natural Hydrogels

Natural polymers such as cellulose, starch, chitosan, peptides/proteins, and their derivatives have been used extensively in hydrogels for sensing applications owing to their widespread functional groups [8] and distinct chemical and physical properties; hence, they are more reliable than its synthetic derivatives. The intrinsic properties of natural materials, such as unique bioadhesion properties, specific environmental responsiveness, superior biocompatibility, and swelling behavior, make them ideal candidates for sensing applications [9,10].

Cellulose is one of the abundantly available renewable materials extensively used in the food, textile, packaging, paper, and biomedical industries. It is generally hydrophobic in nature but insoluble in common solvents and water due to the strong hydrogen bonds between the cellulose molecular chains [11,12]. Recently, nanocelluloses, such as cellulose nanocrystals (CNCs) and cellulose nanofibrils (CNFs) have been used for mechanical reinforcement in hydrogel matrices, due to their high surface area, wettability, and superior moisture absorptivity [13]. Nanofibrils and crystals comprise β (1-4) linked D-glucose units along with a semicrystalline nature and exhibit extensive mechanical properties through surface modifications [14]. The enhanced mechanical strength in cellulose is mainly used for strain sensors [15]. It is reported that CNF-PVA composite-based hydrogel sensors have been used to fabricate expandable, transparent, and self-healing hydrogel sensors [16]. Multiwalled carbon nanotubes (MWCNTs) with cellulose fibers are used to synthesize piezoelectric-based pressure sensors and biodegradable materials, where cellulose fibers exhibit a shaper memory alloy effect and flexibility and the MWCNTs influence the conductivity [17].

**Figure 2:**
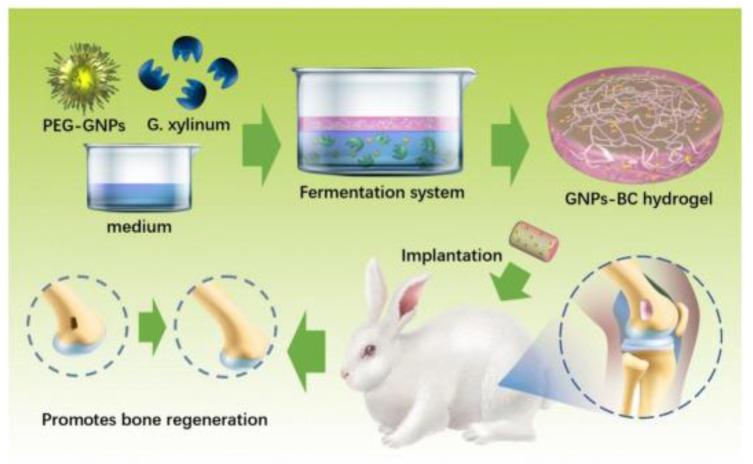
Schematic view of PEGylated gold nanoparticles in fermentation medium containing *G. xylinum* to fabricate GNP-BC hydrogels to promote bone regeneration applications [20]. Copyright received.

Cellulose and nanocellulose-based strain sensors have received considerable attention in reinforcement, where the reinforcement agents will be functionalized to impart sensing characteristics. Nanocellulose and CNCs are extensively used to produce hydrogel-based sensors with superior transparency and mechanical strength. Bovine serum albumin (BSA) encapsulated–gold nanoparticle clusters were assembled with alginate/CNC hydrogels for heavy metal detection [18]. Zhou and Wang stated that cellulose hydrogels are a potential candidate for heavy metal detection processes with fluorescent composite reinforcement [18]. A combination of PVA, CNC, and PVP composite hydrogels was used as soft strain wearable sensors, which evolved self-healing properties, good elastic properties, and better conductivity. In addition, carbon nanotubes and carbon black modified with sodium alginate are also used in strain-sensing applications [19]. Huang et al. naturally synthesized PEGylated gold nanoparticles at different concentrations and treated them with fermentation medium containing *Gluconacetobacter xylinus* (*G.xylinum*) to fabricate in situ Au/BC hydrogels (Figure 2) for bone tissue regeneration applications [20].

**Figure 3:**
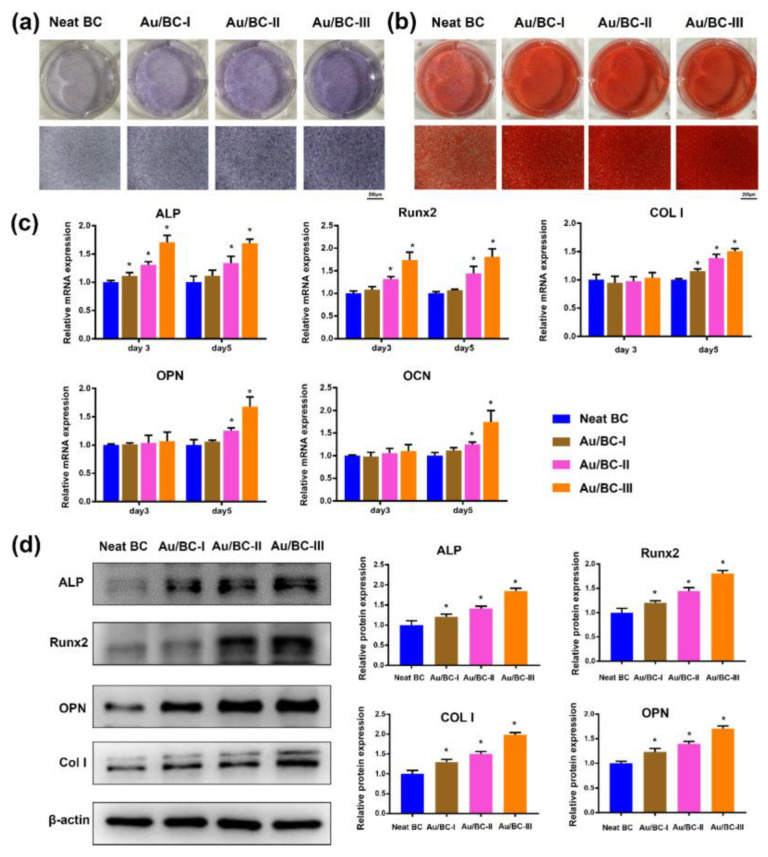
Osteogenic assessment of Au/BC hydrogel. (**a**) ALP and (**b**) alizarin red staining of hBMSCs after 14 and 21 days of treatment with the Au/BC hydrogel. (**c**) RT-PCR analysis of Runx-2, ALP, COL1, and OPN after treatment with liquor extraction of Au/BC hydrogels for 3 to 5 days in the presence of hBMSCs, (**d**) Runx-2, ALP, COL1, and OPN expression by Western blot study of Au/BC hydrogel extraction after 7 days of incubation [20]. Copyright received. * The values are significant.

Huang et al. studied the osseointegration capability of Au/BC hydrogels compared with neat BC hydrogel scaffolds (Figure 3). ALP and ARS staining results illustrated that Au/BC hydrogels triggered higher ALP activity with increased calcium nodules in hBMSCs compared to neat BC hydrogels. Furthermore, mRNA expression levels, including Runx2, COL1, OCN, and OPN, were significantly upregulated in the extraction of Au/BC hydrogels. In addition, Au/BC-III hydrogels significantly improved osteogenic differentiation [20]. Cellulose derived from bacteria exhibits a highly pure form in nature and can be reinforced with hydrogels owing to its crystallinity and ultrafine structure, thereby providing durable and high mechanical strength to hydrogel sensors [19]. Since hemicelluloses, lignin, and pectin do not exist in bacteria, cellulose in its pure form is more suitable for hydrogel sensors, especially in medical applications [13].

Starch is an important natural polysaccharide that comprises several glucose molecules or monosaccharide units joined with α-D-(1-4) or α-D-(1-6) linkages, which contain several hydroxyl groups used for hydrogel preparation with chemical modifications [21]. Starch exhibits intensive expanding ability and has been used in heavy metal capture, dye sorption, and monitoring water quality in agriculture [22]. Starch-based hydrogels are used to stabilize silver nanoparticles for surface plasmon resonance (SPR) sensors for the detection of hydrogen peroxide (H_2_O_2_).

### 2.2. Synthetic Hydrogels

Synthetic polymers including PVA (polyvinyl alcohol), PEG (polyethylene glycol), and PAM (polyacrylamide) have intrinsic characteristics such as flexibility, desired mechanical strength, controllable molecular structures, and selective mechanical properties, especially when used for sensor and actuator applications. PVA possesses unique biocompatibility, mechanical properties, and biodegradation performance [23]. It exhibits ultrahigh absorbance and excellent solubility, which leads to metal ion sensing applications. It also contains a significant amount of hydroxyl groups that allow modifications in PVA polymer chains. The crosslinking of PVA chains can be performed by either physical or chemical modifications.

PEG is another important polymer that is significantly used in biomedical industries. PEG is a well-known material studied for different applications including glucose, chemical, and gas sensing applications [24]. PAG (polyalkylene glycol)-based hydrogels have received more attention than PVA- and PEG-based hydrogels due to their superior biocompatibility, biosensing, and well-defined methodology for the fabrication of robust platforms for desired physiological and mechanical performances [25]. Double-network PAM hydrogel sensors are mainly used for strain and pressure sensors, which can be further developed into wearable sensors [26]. Hou fabricated a high-performance injectable PAM/polysaccharide-based ionic hydrogel network for strain sensing applications [27]. Photonic crystal-based PAM hydrogel systems are mainly used to detect glucose or pH by tracking the ionic strength of photonic crystals [28]. In addition, proteins, peptides, antibodies, or enzymes were immobilized with PAM hydrogels, which have been increasingly used in molecular detection systems [29]. PAM in composite form is effectively used as a humidity sensor due to its high absorbance ratio.

### 2.3. Functional Polymers

Stimuli-responsive polymers have received increasing attention for various sensing applications based on visible/ultraviolet (UV/Vis) [30], electric field [31], mechanical stimuli [32], magnetic field [33], biological [34], and chemical-based stimuli responses [35]. The reinforcement of functional polymers into hydrogels has found various applications in sensors, actuators, drug delivery, and scaffold systems [36].

Markstedt et al. fabricated an ink from alginate/nanocellulose composites crosslinked with Ca^+^. Alginate hydrogels tend to have loosely bound structures when placed in a Ca^+^ medium. Similarly, the same group developed xylan/nanocellulose composite hydrogels, where the content of nanocellulose was reduced to one-third or even less [37].

**Figure 4:**
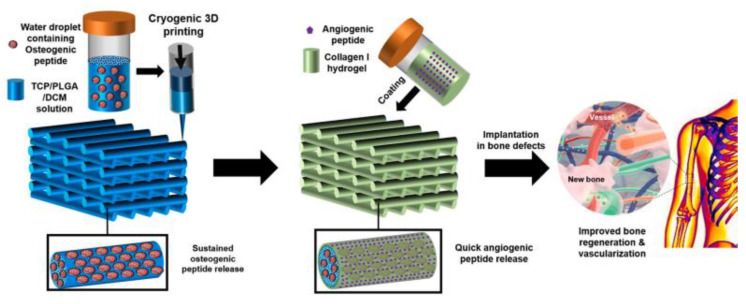
Schematic representation of the synthesis of OP and AP delivered PLGA/TCP nanocomposite 3D-printed scaffolds using a cryogenic 3D printing process and hydrogel coating [38]. Copyright received.

Wang et al. developed a customized composite scaffold that constitutes TCP/OP/PLGA struts along with collagen I coating with the aim of a dual delivery system developed by cryogenic 3D printing technology for bone regeneration applications (Figure 4). Nanocomposite-coated collagen I scaffolds provide better angiogenesis with the capability of quick release of AP followed by AP release in a sustained manner. The 3D-printed constructs improved bone and vascularization growth through local drug delivery [38].

**Figure 5:**
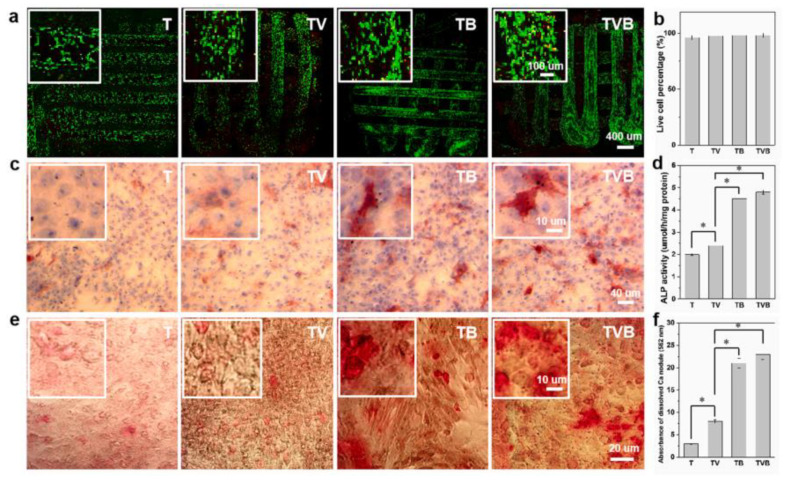
Osteogenic differentiation ability of rBMSCs cultured with scaffolds. (**a**,**b**) Live/dead cell analysis after 3 days of rBMSC culture; (**c**,**d**) ALP staining and activity after extraction with scaffolds; (**e**,**f**) Alizarin Red S staining with quantitative evaluation of calcium nodules [38]. Copyright received. * The values are significant.

The osteogenic capability of the scaffolds in vitro was studied using rBMSCs as model cells. The scaffolds exhibited 98% live cells, confirming their biocompatibility after 3 days of incubation. After 7 days of culture, significant ALP^+^ levels were improved, illustrating the osteogenic differentiation property (Figure 5). In addition, after 21 days of incubation, the TB and TVB groups were significantly improved, which favors more calcium precipitation, indicating the release of both OP and AP from the scaffold area for better osteogenic differentiation.

### 2.4. Nanocomposite Hydrogels

In recent decades, interest in combining nanotechnology with other fields of study has significantly increased. Many attempts have been made to merge nanoscale procedures with regular methods to fabricate better materials. Nanocomposite hydrogels are an example of such a combination of nanotechnology and biomaterials science [39].

**Figure 6:**
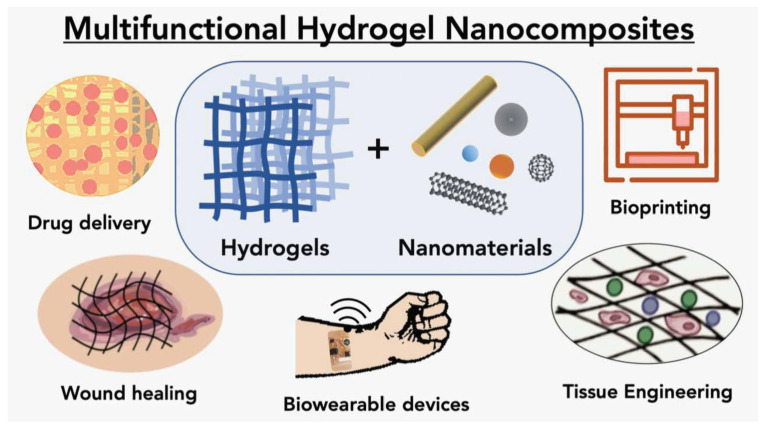
Schematic representation of multifunctional hydrogel nanocomposites [40]. Copyright received.

Barrett-Catton et al. stated that nanocomposite hydrogel materials play a significant role in many applications, such as targeted drug delivery applications (Figure 6), tissue engineering, bio-wearable devices, and bioprinting applications [40]. Hydrogels are hydrophilic materials with the macromolecular structure of a gel that is made up of interlinked natural-synthetic polymer chains joined together by crosslinkers [41,42]. They have the ability to expand to many times their dry weight and hold up to 99% water or biological fluids. This three-dimensional (3D) hydrated porous network is often designed to absorb, release, or immobilize biological entities and can replicate the original tissue microenvironment [43]. Today, the use of these systems as drug delivery platforms has been reduced in many circumstances due to weak mechanical characteristics, relatively rapid drug release from the gel, and inefficient hydrophobic drug loading [44,45].

**Figure 7:**
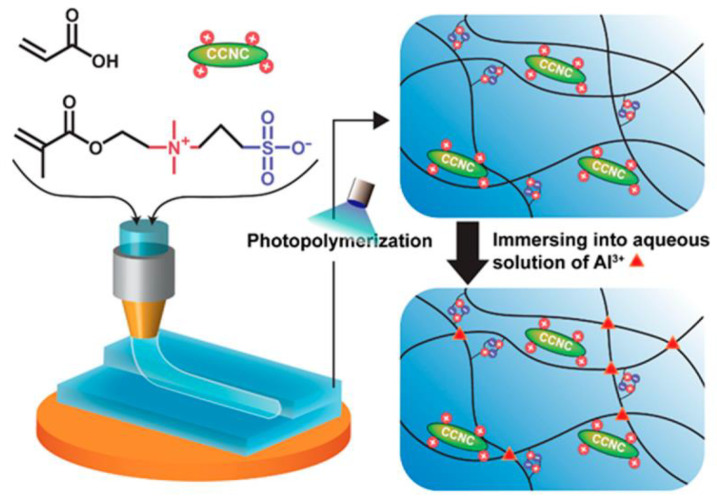
Schematic illustration of 3D-printed CNC-reinforced zwitterionic hydrogels for strain sensors [48]. Copyright received.

Various types of nanoparticles, including metal and metal oxide nanoparticles (silver, gold, iron oxide), inorganic/ceramic nanoparticles (hydroxyapatite, silica, silicates, calcium phosphate), polymeric nanoparticles (hyperbranched polyesters, cyclodextrins, etc.), and carbon-based nanomaterials (carbon nanotubes (CNTs) and graphene) are mixed with polymer networks to construct reinforced polymer hydrogels and build nanocomposites with specific physical characteristics and functions [46,47]. Lai et al. prepared an unmodified cellulose nanocrystal (CNC)-based ink for making 3D-printed strain sensors and obtained highly stretchable and mechanically stable sensors. However, they could not achieve transparency. Hence, they added acrylic acid, 3-dimethyl (methacryloyloxyethyl) ammonium propane sulfonate (DMAPS) into CNCs and obtained a highly transparent (Figure 7) sensor without compromising the mechanical and stretchable properties [48].

The nature of the nanoparticles incorporated into the hydrogel governs the kind of stimuli that may be utilized to release the drugs under the optimal conditions and also enhances transport to different sections of the body, allows for the administration of hydrophobic pharmaceuticals, or is responsible for multistimuli-response systems [49]. Furthermore, certain properties are improved in the nanocomposite hydrogel, including mechanical properties, adhesion to surfaces, and the ability to produce microfabricated structures [39,50].

### 2.5. Conducting Hydrogel

Electrically conductive hydrogels (ECoHs) are promising prospects for bioelectronic applications such as drug delivery systems in skin and muscle and monitoring the heart and neurons [51]. Due to their mechanical characteristics, bioactivity, water retention, and other extracellular matrix-like features, ECoHs have been at the forefront of developing smart conductive biomaterials [52]. ECoHs have made it possible to minimize property mismatch at bioelectronic interfaces, which is difficult to achieve on a conventional electronic interface [53]. In addition to the innovation of novel hydrogel systems, 3D printing of such ECoHs is one of the most sophisticated methods for swiftly fabricating future biomedical devices and implants with flexible designs and customizable functionality.

**Figure 8:**
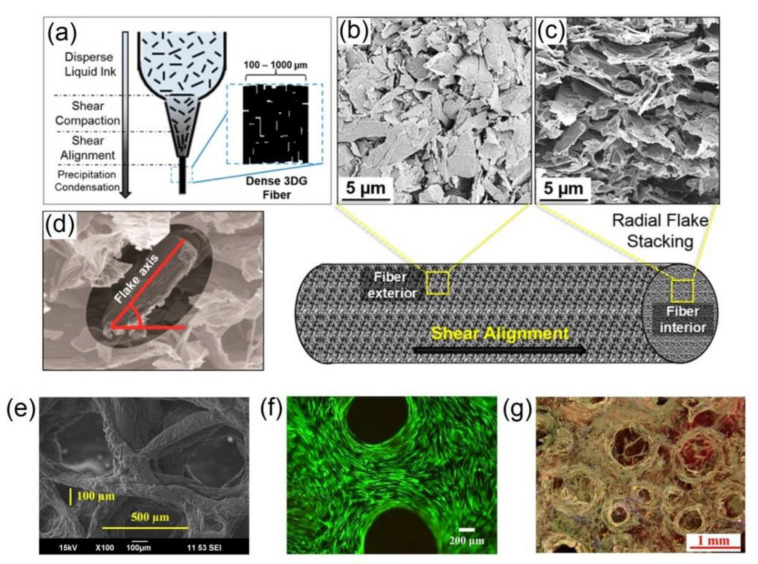
Schematic view of (**a**) 3D-printed composite hydrogel showing graphene flake; (**b**,**c**) surface morphology and cross-sectional view of graphene; (**d**) graphene flake orientation; (**e**–**g**) SEM, fluorescence, and light microscopy images of composite hydrogels [55]. Copyright received.

Bioplotting, light-based techniques, and inkjet printing are examples of basic 3D printing concepts. Conductive hydrogels are deposited through a nozzle onto a substrate of interest (e.g., a tissue culture well plate) in 3D bioplotting. A major problem is ensuring the dimensional stability of the printed structures, which can be done, for example, by recovering the previously sheared hydrogel or immediately crosslinking the printed structure after printing [54]. Jacus et al. fabricated ECH nanocomposite structures (Figure 8) with superior electrical conductivity (875 S cm^−1^) and good biocompatibility with human mesenchymal stem cells [55]. A photoreactive hydrogel precursor is illuminated with either a moving UV light or laser beam or with a pattern projected onto the hydrogel precursor to induce curing in specific regions to form meshed patterned areas in light-based printing approaches such as stereolithography (SL/SLA), direct laser writing (DLW), or DLP. The precursor reservoir is frequently z-shifted to facilitate layer-by-layer deposition in the z-direction to generate 3D structures. Microvolumes of either conductive hydrogel are deposited onto a printing stage using inkjet printing by deforming a piezo element, resulting in the deposition of microscopic droplets [56].

With the growing quantity of literature, it is obvious that researchers have begun to recognize the benefits of and difficulties in 3D printing. The primary functionalization strategies for achieving electrical conductivity in hydrogels were given, and they were identified as composite approaches utilizing conductive particles. The primary uses of hydrogel materials in conjunction with 3D printing technology include flexible electronics, tissue engineering, and biosensors, as detailed in Table 1.

### 2.6. Photoresponsive Hydrogels

**Figure 9:**
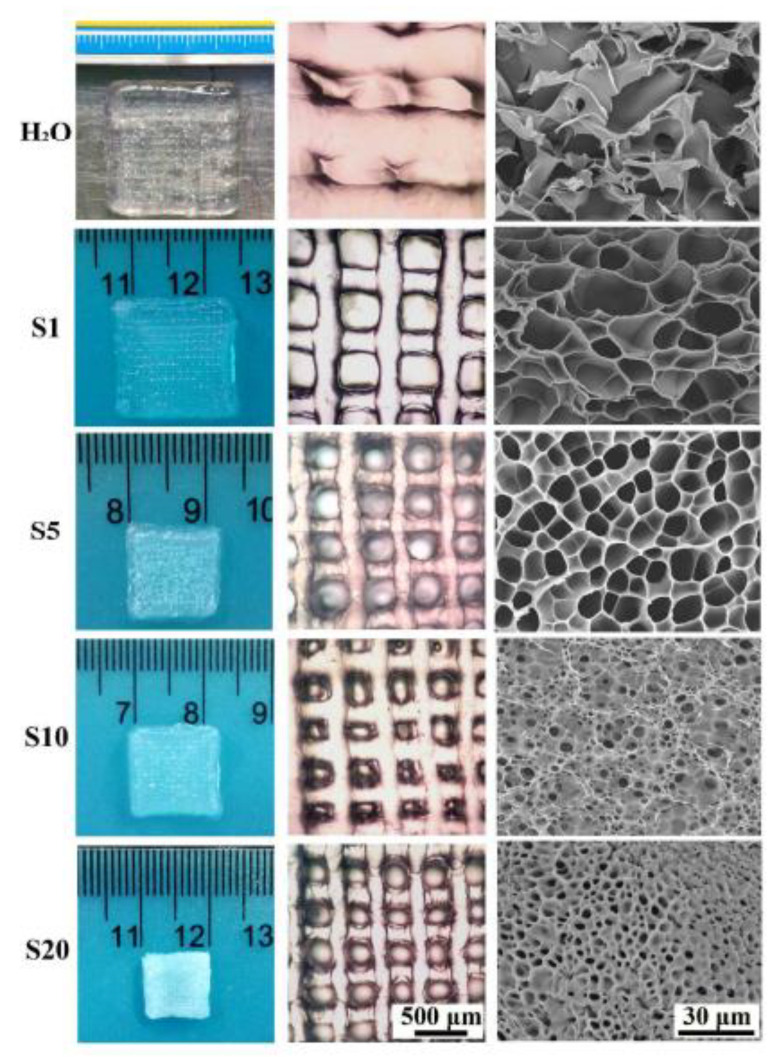
Photographs, microscopic and SEM images of 3D-printed HBC hydrogel scaffolds treated with H_2_O as well as different concentrations of NaCl solutions [65]. Copyright received.

A polymeric network and a photoreactive moiety, generally a photochromic chromophore, make up photoresponsive hydrogels. They are intelligent hydrogels that may alter physically or chemically in response to an optical input. Photosensitive materials have three response modes. When the internal temperature of a gel meets a phase transition criterion, the gel produces a reaction. Photoresponsive molecules in temperature-sensitive materials transform light energy into thermal energy and raise the temperature of the substance [63]. To respond to light exposure, photosensitive molecules generate ionization inside a gel. When the gel is exposed to light, a huge number of ions are produced within it, disrupting the osmotic equilibrium, which is compensated for by water molecules and drifting ions. Photochromic molecules are inserted into the hydrogel material as pendant groups or crosslinkers on the polymer backbone. Because of their light sensitivity, these chromophores’ physicochemical characteristics, such as dipole moment and geometric structure, as well as the form, structure, and properties of the macroscopic hydrogel, are affected by swelling and contraction [64]. Wang et al. constructed mechanically stable multilayer hydroxylbutyl chitosan (HBC) hydrogel scaffolds by a 3D printing process. After scaffold preparation, different NaCl concentrations were used to determine the swelling efficacy of the HBC scaffold (Figure 9). From the investigation, they observed that the scaffold structure rapidly swelled and loosened its structure with increasing NaCl concentration [65].

Natural or synthetic polymers must meet two criteria to be employed in hydrogels: a high molecular weight backbone or side chain with a significant number of hydrophilic groups and an acceptable crosslinked network structure. A monolithic water-soluble or oil-soluble monomer, a polymeric natural or synthetic polymer, or a combination of a monomer and a polymer can be used to make the smart hydrogel material. Photochemical reactions that govern spatial, temporal, and dosage control may be achieved utilizing biophysical and chemical signals, providing strong tools for simulating the complex milieu of the ECM and regulating cell activity. Photochemical reactions now need the inclusion of a photoinitiator or the formation of organic byproducts during photolysis, potentially bringing biotoxicity into the 3D cell microenvironment [66].

De Forest and colleagues reported a reversible technique that linked thiol-ene photocoupling with the photodegradation of an o-nitrobenzyl group, allowing them to create better strategies for stem cell culture, 3D cell culture tests, and tissue regeneration [67]. Ma and his team fabricated a light-sensitive hydrogel microsphere based on cyclodextrin and azobenzene host-guest interactions. To enhance the number of cells cultivated on a surface, the photosensitive hydrogel was made of PEG-crosslinked poly (methyl vinyl ether-alt-maleic acid) and cyclodextrin (PMVE-alt-MAH—CD) linked to azobenzene arginine glycine aspartate (azo-RGD) through host-guest interactions. When exposed to UV light, the azo-RGD separated from the hydrogel, resulting in a reduction in the cell population [68].

### 2.7. Synthetic Hydrogels

Hydrogels are one of the key areas of study for biomaterials research and have a three-dimensional polymer network. Due to the presence of hydrophilic groups in their structure, they have the ability to absorb significant volumes of water or biological fluid in the body. Hydrogels can be categorized into two groups, natural hydrogels and synthetic hydrogels, depending on the kind of polymer used [41]. Natural polymers, including polysaccharides and their associated proteins, are frequently utilized as carriers for releasing chemical substances. Natural hydrogels, including alginate, collagen, gelatin, and fibrin, are utilized in clinical applications. Synthetic hydrogels are hydrophobic. They outperform natural hydrogels in terms of chemical composition and mechanical structure [69]. These polymers include PVA, PEG, and PAA and its derivatives. One of the most widely used synthetic hydrogenation polymers is PEG, which is employed in a variety of medicinal applications including medication administration, bone prostheses, tissue engineering, and wound dressings. Synthetic hydrogels are utilized for various medical applications due to their properties, such as resistance to protein adsorption, non-stimulation of the immune system, and biocompatibility [70].

Synthetic polymers are used to produce synthetic hydrogels. The polymerization of a monomer yields these man-made polymers, including polyethylene glycol (PEG), polyvinyl alcohol (PVA), polyethylene oxide (PEO), poly-N-isopropyl acrylamide (PNIPAM), poly-2-hydroxyethyl methacrylate (PHEMA), polyacrylamide (PAAM) and polyacrylic acid (PAA). Some, such as PAAM, are biocompatible and are stable and mechanically strong [71]. In 3D cell cultures, synthetic hydrogels may support different cell types structurally due to their strong mechanical properties. Due to their biocompatibility (adaptability and nontoxicity for live tissue), lack of immunogenicity, and stiffness, these PVA and PEG hydrogels are commonly utilized for 3D scaffolds [72].

According to Wilkinson et al., PVA hydrogels can replace albumin to promote the expansion of murine hematopoietic stem cells (mHSCs) [73]. Growth factor-infused PVA hydrogels have been shown to increase the pace at which mouse spermatogonia stem cells (mSCCs) differentiate into meiotic and postmeiotic cells [74]. Additionally, in 3D cultures using cell plates coated with PVA-hydrogels, a number of human glioma cell lines, including LN299, U87MG, and Gli36, may create tumor spheroids [75]. The 3D cultivation of human pancreatic cancer cell lines (Sui67 and Sui72) and human breast cancer Hs578T cells with PVA hydrogels produced results that were comparable. This finding demonstrates that PVA hydrogels can inhibit cancer cell apoptosis and stimulate cancer cell growth [76].

Both natural and synthetic hydrogels have demonstrated several benefits in 3D cell cultures, but they also have certain drawbacks. While manufactured hydrogels are physiologically inert and devoid of endogenous components, natural hydrogels have a quick breakdown, weak stability, and low mechanical strength. Natural and synthetic polymers are combined to create semisynthetic hydrogels. In 3D cell cultures, the combination reduces the drawbacks of natural and synthetic hydrogels. The chemical and physical characteristics of synthetic polymers may be easily changed. The most competitive materials for the manufacture of intelligent hydrogels for drug administration are synthetic hydrophilic and biodegradable polymers. Smart hydrogels are enhanced by these synthetic polymers to have low toxicity, minimal side effects, and minimal blood material adherence. Low blood material adherence can decrease the impact of opsonization and lessen phagocyte clearance [77].

#### Limitations

The main drawback of synthetic hydrogels includes a lack of cell-specific bioactivities, such as cell adhesion, migration, and cell-mediated biodegradation. One of the drawbacks of 3DP is that the pore diameters of manufactured scaffolds rely on the powder size of the stock material. As a result, the range of pore sizes is restricted to lower pore values dispersed across the scaffold. Prior to the creation of the scaffold, porogens (of predetermined sizes) can be mixed into the powder to produce pores of a wider range of sizes. However, partial leaching is occasionally observed, leading to incomplete elimination of the porogens. The mechanical properties and accuracy of 3DP-fabricated scaffolds are other considerations that need to be addressed [78].

## 3. 3D Printing Process

### 3.1. Inkjet Laser 3D Printing

**Figure 10:**
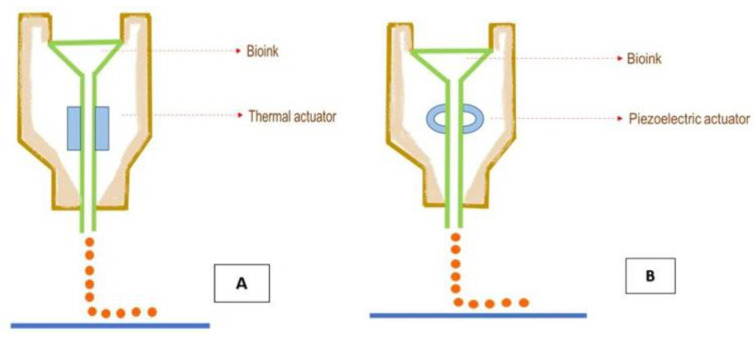
Schematic diagram of inkjet-based bioprinting using (**A**) thermal and (**B**) piezoelectric actuators.

Droplet-based bioprinting uses acoustic, thermal, or electric fields to print bioink in a layer-by-layer approach (Figure 10). This bioprinting method can be further classified into four types, namely, inkjet printing, acoustic printing, microvalve printing, and electrohydrodynamic printing [1]. Inkjet printing had its origin in the 1950s with the invention of the inkjet device by Elmqvist of Siemens [79]. This noncontact process involves dispensing the bioink that contains cells along with growth factors onto a substrate [80]. This technology is highly used compared to others because of the ease of use, adaptability, excellent control over the deposition pattern, and formation of heterogeneous cell constructs with defined positions [81,82]. It has been reported that bioink with a viscosity of less than 10 mPa/s can only be produced using this method. Inkjet printing can be further classified into three working modes: continuous inkjet bioprinting, electrohydrodynamic jet bioprinting, and drop-on-demand bioprinting [83]. Low-viscosity bioink passes through the nozzle and flows as droplets in continuous inkjet bioprinting. Drop-on-demand bioprinting involves droplets of bioink flowing out of the nozzle with the help of piezoelectric or thermal actuators [84]. Thermal drop-on-demand-based bioprinting employs heat, whereas piezoelectric-based bioprinting employs acoustic pulses generated when electric stimuli are applied to a piezoelectric material for the ejection of bioink [85]. The droplet size can be regulated between 1 and 300 pL and the deposition rate can range between 1 and 10,000 droplets per second [86]. The main advantages of inkjet-based bioprinting are its capacity to regulate droplet size, homogeneity, and directionality, as well as its faster printing rate and affordability [87].

#### Challenges

The main problem with inkjet-based bioprinting is nonuniform droplet formation, deposition location that is not accurate, and cell activities damaged by heat. Another main hindrance to using this method is the requirement for low-viscosity bioinks [88]. Limitations in software and hardware also prevent the usage of diverse cell types. Recurrent nozzle clogging prevents the use of heterogenous bioinks. Printing height and cell viability greater than 85% are also challenges faced during inkjet bioprinting [89].

### 3.2. Extrusion-Based 3D Printing

**Figure 11:**
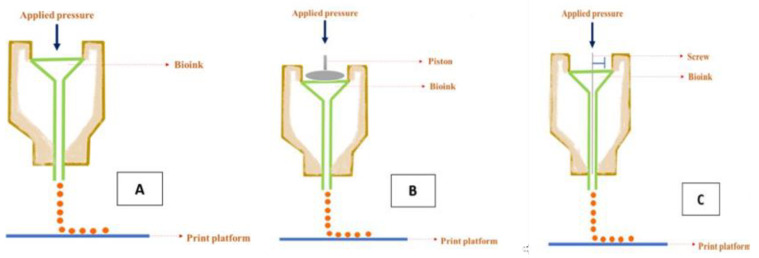
Schematic diagram of extrusion-based bioprinting using (**A**) pneumatic-, (**B**) piston-, and (**C**) screw-based methods.

Crump was the first to introduce extrusion-based bioprinting [90]. This method makes use of mechanical or pneumatic potential energy to extrude the bioink through a nozzle in cylindrical filaments (Figure 11). It is likely to be the most extensively used 3D bioprinting technology because of its excellent flexibility to accommodate a wide range of fluids with viscosities of between 30 mPa s^−1^ and about 6 × 10^7^ mPa s^−1^ [91]. This technique delivers higher cell densities than inkjet bioprinting but at a lower speed and resolution [92]. The mechanism behind the printing process is based on the continuous application of air pressure, which causes the bioink to be extruded as an uninterrupted cylindrical filament from the needle head or nozzle [93]. Pneumatic-based extrusion, piston-based extrusion, and screw-based extrusion are the three categories of extrusion techniques based on the extrusion mechanism [94]. Pneumatic-based extrusion is based on air pressure and the remaining two modes are mechanically based [95]. The extrusion rate can be easily altered in pneumatic-based extrusion systems and it is highly preferred for low-viscosity solutions [96]. High-viscosity bioinks can be easily extruded using piston-based extrusion and the rate of extrusion can be altered by controlling the movement of the motor [97]. In screw-based extrusion systems, the raw material is put into the cartridge and pushed by a motor-driven auger screw, and the ink emerges via the extrusion nozzle [96]. The main advantages of this method are that it is simple and affordable. In addition, a wide range of materials can be printed and required hardware and software upgrades can be performed very easily [98].

#### Challenges

The main drawback of extrusion-based bioprinting is that it can be employed only for viscous liquids. The printing speed and resolution are also very slow when compared to other bioprinting methods. In addition, nozzle clogging can disrupt the formation of the 3D structure [99].

### 3.3. Digital Light Processing (DLP) 3D Printing

**Figure 12:**
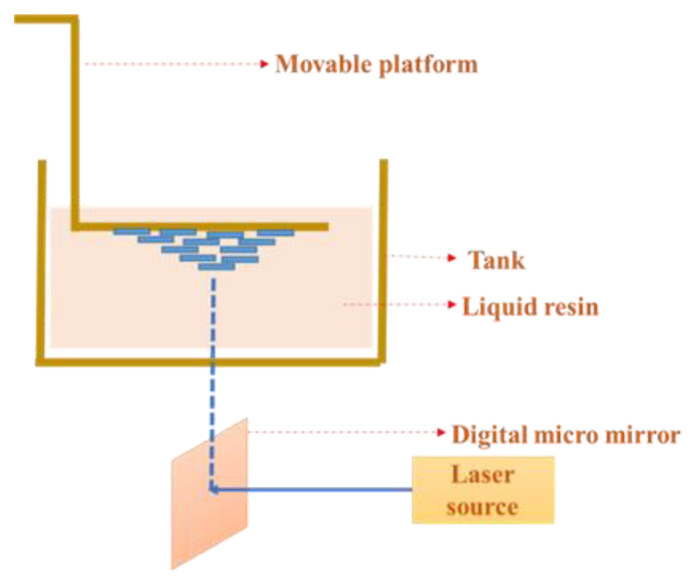
Schematic diagram of digital light processing bioprinting technique.

DLP 3D printing is a light curing-based method that creates a pattern of light using a digital micromirror device (DMD) and helps to create complex features [100]. This method employs photocurable resins that are appealing among the many 3D printing techniques (Figure 12), and it is utilized to construct a single layer of the 3D structure by spatially controlled solidification using a projector lamp (mostly UV or blue light). Photocurable resins are unsaturated polyesters that solidify upon exposure to light. The properties of the printed structure can be altered by varying the photocurable resin [101,102]. It is possible to produce a vast variety of systems for the construction of scaffolds with complex characteristics and functions [103]. The DMD array receives incident light from the lamp. The orientation of each individual mirror is adjusted by a graphical processing unit (GPU) by reading the input digital mask and applying a potential to the DMD array via a controller. The DMD array reflects light, and the intensity of that light in the projection direction depends on the orientation of the mirrors [104,105]. The DMD is also referred to as the dynamic mask that helps in the projection of light [86]. The resolution is usually dependent on the light source and offers higher accuracy compared to other types of bioprinters [106]. The main advantages of this method include high print speed and high precision, and the fact that it can be used for various applications [107].

#### Challenges

The main drawback of DLP-based printing is that it can only print small objects. In addition, the printer is very expensive since the technology is proprietary [108]. Due to the high precision of DLP 3D printing technology and the fact that it can only print small-scale models, it is highly utilized in the dental and jewelry casting industries [109].

### 3.4. Stereolithography (SLA) 3D Printing

**Figure 13:**
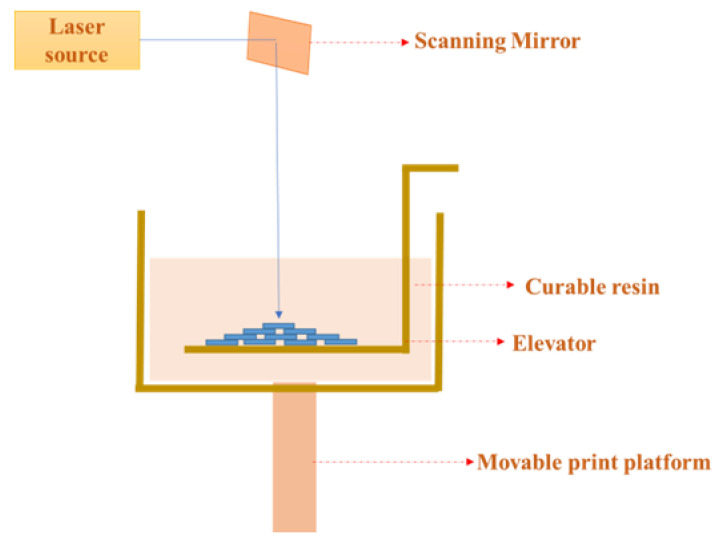
Schematic diagram of stereolithography bioprinting technique.

Stereolithography (SLA)-based 3D printing (Figure 13) is a light-curing method that utilizes a laser that moves automatically [110]. A photosensitive resin is used, and this is the only photocuring method that can be used to print large objects since the laser beam can pass through large spaces [108]. One of the main components of the stereolithography process is the photocrosslinking reaction where the photo-initiators break down to form free radicals and start the reaction [111]. UV light is projected based on the structure to be printed onto the surface of the bioink. This method is used to print intricate structural designs and takes the same amount of time and has a high level of accuracy compared to other printing methods [110]. The illumination source has an effect on the resolution as well as the accuracy of the objects to be printed. The mechanical properties can be varied by altering the source of light [112]. The 3D model to be printed is sliced into 2D images. The bioink is printed up to the desired thickness. The 2D stack image given in the input acts as the mask and is projected into the bioink, thereby initiating the curing process [113]. The main advantages of this method include multi-scalability, very high resolution, and quick printing of extremely complex scaffolds. The printer is very easy to use and control and thus highly preferred [114]. Table 2 presents a comparison of different 3D printing techniques.

#### Challenges

One of the major disadvantages of this printing process is the poor printing rate because of its dependency on the curing rate. The printing rate decreases as the size of the printed object increases [115]. The resolution in this technique is limited compared to other photocuring methods since the size of the laser beam spot depends on the laser source [116]. In some cases, 3D structures printed through the SLA method are toxic to cells by reducing the viability of the cells [117].

**Table 2:** Comparison of different 3D printing techniques.

Process	Advantages	Disadvantages	Resolution	Cost	Speed	Viscosity	Ref.
Inkjet	Capacity to regulate the droplet size, Homogeneity, Directionality as well as its faster printing rate, Affordability, Low cost	Nozzle clogging, Non-uniform deposition, Viability of the cells should be greater than 85%	50 μm	Low	Slow	Less than 10 mPa/s	[91,116,118]
Extrusion	Simple, Affordable, Wide range of materials can be printed	Viscous liquids are needed, Printing speed is low, Cell viability is low	100 μm	Medium	Fast compared to inkjet printing	30 mPa/s to 6 × 10^7^ mPa/s	[91,117,119]
Digital light processing	High print speed, High precision, High Resolution	Only small objects can be printed, Cost is high compared to other printing methods	20–200 μm	Very expensive Process	Speed varies according to the source of the laser beam and the size of the model to be printed	Nil	[117,118,119,120]
Stereolithography	No nozzle required. High accuracy Easily print complex structures	Resolution is limited, Cell toxicity, Reduced printing rate	100 μm	Low	Speed varies according to the curing rate and size of the object to be printed	1 to 300 mPa/s	[45,91,119]

## 4. Applications of Hydrogels in 3D Printing

Three-dimensional (3D) printing technology based on hydrogels has evolved as a viable treatment alternative for bone and joint injury, and its full potential is just now being realized. The bulk of the healing properties of existing repair materials are present in 3D-printed hydrogel materials, but they also offer outstanding ductility, hydrophilicity, and histocompatibility. Therefore, hydrogels have tremendous potential for repairing bone and joint tissue, fighting infection, and even treating tumors due to their unique benefits [121].

Bone has different qualities to cartilage and so has different needs for 3D-printed hydrogel materials, including certain higher-level functions and features [122]. For example, 3D-printed hydrogels must be stronger and harder than cartilage in order to heal and regenerate bone tissue [123]. In addition, 3D-printed hydrogels must have the mechanical strength to preserve not only human mechanics but also the mechanical stability of the 3D-printed structure itself [124]. Angiogenesis in bone tissue is more essential than in cartilage tissue [125]. The bone tissue structure is complicated, consisting mostly of diverse skeletal cells and interconnected blood arteries. Blood arteries provide the oxygen and nourishment that bone tissue cells require. As a result, pro-angiogenic characteristics of bone tissue are required for 3D-printed hydrogels.

Currently, 3D-printed hydrogels play an important role in the medical research field. Hydrogels are primarily used in drug delivery and nerve damage repair. Nerve guidance conduits (NGCs) can connect the proximal and distal ends of defective nerves. The ideal NGC has sufficient mechanical strength and is electrically conductive and biocompatible [126]. In recent years, 3D-printed hydrogels derived from natural and synthetic materials (alginate, agarose, chitosan, degradable polyurethane, etc.) have been increasingly used to serve as nerve guidance conduits, which are the major advantages in the biomedical industry. The extrusion printing method has been widely used in fabricating 3D hydrogel structures because of its unique property of being mechanically stable and producing complex shapes [127]. Johnson et al. synthesized 3D-printed static nerve hydrogels for making sensory and moto branch structures using extrusion printing technology [128]. Cui et al. developed bilayer PU/CoINGC by the FDM technique to make the outer portion of NGCs with a highly oriented fiber structure [129]. Similarly, Hu acquired human sciatic nerve data using magnetic resonance imaging and produced customized methacrylate gelatin NGC for mimicking human sciatic nerves [130]. Zhu et al. synthesized conductive catheters for vascular regeneration and peripheral nerve growth applications [131].

Currently, 3D-printed hydrogels are one of the most common materials for bone tissue growth, and their antibacterial and anti-infective capabilities are outstanding. Furthermore, research into the antibacterial characteristics of hydrogels is currently one of the most prominent study areas. Infection and inflammation cause a major amount of articular cartilage and bone loss, and infection can cause irreparable injury to bones and joints. Other causes of osteoarthritis damage, such as external mechanical stress, can result in secondary infections. The problem of infection will continue throughout the process of rebuilding and repairing bones and joints since there are several medical procedures, such as cartilage and bone repair and reconstruction, and because the implants themselves can lead to infection. Hydrogel materials are currently being researched to study their antibacterial potential and anti-infective qualities [132].

Antimicrobial metal ion nanoparticles such as Ag, Cu, and other metal ions, and ZnO, NiO_2,_ and other metal oxides have been incorporated into hydrogel materials. Hydrogel materials that contain antibiotics and antimicrobial agents are primarily mixed with hydrogel and covered in the hydrogel material, relying on the hydrogel’s liquid behavior to be released at the appropriate time to play antibacterial and anti-infective roles [133]. While hydrogel materials that contain antibacterial and anti-infective properties are uncommon, they have clear advantages; they are bactericidal and effective, with no hazardous side effects.

### 4.1. Limitations

In addition to the range of hydrogel characteristics and the mechanical strength of hydrogels, there are a few more issues to consider. Hydrogel mechanical strength consists of compressive strength, tensile characteristics, and shear thinning capabilities. To begin, the 3D-printed hydrogel bio-skeleton should be able to maintain its own shape and structure while also resisting external stresses. Furthermore, the hydrogel may be stretched and pulled during the 3D printing process, and the ductility of the material with compressibility may be less than optimal. A hydrogel material with significant and comprehensive capacities to deal with varied experimental and clinical conditions is required for practical clinical applications [121].

It is also important to highlight the previously mentioned sterility and antibacterial capacity. Adding metallic or antibiotic substances that are also antimicrobial to hydrogel materials is the primary strategy for addressing the antimicrobial problem. However, because these substances are added, they also have the same long-term release and even distribution issues as the cytokines mentioned earlier. We must assure the sterility of the 3D-printed hydrogel material throughout the process, not just in the experimental setting, in addition to its antibacterial qualities. To ensure the sterility of the 3D-printed implant material, the process flow of the manufacture and the surgical self-contained complete procedure must be designed according to the unique features of the hydrogel for practical applications [134].

### 4.2. Maxillofacial and Craniofacial Applications

Craniofacial tissues are organized in a sophisticated 3D architecture that comprises facial and cranial bones that give structural support and projection for the soft tissues that lie beneath. These bones are generated by either intramembranous or endochondral ossification and have an inorganic/organic matrix. Mature bone is osteonal, with Haversian systems characterized by concentric matrix lamellae with osteocytic lacunae. The cartilaginous component, however, is made up of chondroblasts and chondrocytes [135,136].

Modern 3D bioprinters can produce very accurate bespoke bone and cartilaginous scaffolds based on individual abnormalities. The high incidence of bone loss due to trauma, osteoporosis, tumors, etc., renders bone to be one of the most frequently transplanted tissues other than cartilage [137]. For such replacements to be successful, these substitutes must use biomaterials including bioceramics, polymers, their composites, and hydrogels that are biocompatible, printable, osteoconductive, osteoinductive, and have equivalent mechanical characteristics [138]. Bioceramic scaffolds implanted in load-bearing parts of the craniofacial region were shown by Saijo and colleagues to be too brittle for implantation. HA/TCP composite scaffolds lacked strength and dimensional stability, especially in maxilla-mandibular deformities [139].

Polymers are the preferred material for bioprinting craniofacial tissues due to their superior printability and effective osteogenesis stimulation. However, they lack the necessary rigidity and show poor cellular contact. Despite being highly popular in the past, PLA and PGA (Polyglycolic acid) are rarely the materials of choice for bone scaffolds because of their low compressive strength and osteoconductivity. Copolymer PLGA, however, was discovered to have outstanding osteoconductivity and superior mechanical characteristics [140]. The Young’s modulus obtained resembles that of real bone. As sought for load-bearing bone and craniofacial implants, high-performance polymers such as polyaryletherketones (PEAKs) are becoming more and more attractive as alternatives for craniofacial tissue. In terms of mechanical strength and biocompatibility, Polyetherketoneketone (PEKK) performs more admirably than other members of the PEAK family. Using PEKK in SLS bioprinters, Adamzyk and colleagues created implants for a model of a craniofacial bone deformity. The resulting structures showed increased biocompatibility, mechanical strength, and osseointegration that was noticeable in vivo. Lin et al. reported similar results using mesenchymal stem cells generated from TMJ synovial fluid [141,142]. A short summary of different bioprinting techniques is displayed in Table 3.

#### Limitations

The principal challenges with this approach may be divided into three categories: material, manufacturing, and vascularity. The selection of the proper biomaterial is the most critical step for the successful bioprinting of therapeutically relevant tissue structures. Many conventional biomaterials are physiologically active enough to generate undesired cellular interactions, resulting in premature or undesirable stem cell development [143]. Despite their non-structural similarities to real tissue and similarity in ECM and other components, the new biopolymers and hydrogels lack structural integrity and are unsuitable for standard bioprinting techniques [144]. As a result, it is advised to mix two distinct biomaterials to maximize their benefits and obtain a hard scaffold with greater mechanical qualities, as well as a softer substance with better proliferative and cytocompatible effects on the build. The lengthy time needed for bioprinting, the challenge of reliably supplying the number of cells needed for tissue regeneration, changes in cellular form, and even cell death are further drawbacks. As a result, the total efficiency of the bioprinting process must be increased [145]. The formulation of vasculature required for tissue life is the other major hurdle in bioprinting functioning tissues. In vivo tissue development beyond 100–200 m requires a vascular network since oxygen can only diffuse within this limit [146]. In the absence of vasculature, freshly produced tissue constructions are deprived of nutrition, resulting in incomplete tissue development or necrosis. It is worth noting that the vasculature must form at an early embryonic stage to prevent tissue death and allow endothelial adhesion and proliferation. Later, the vasculature must take up all of the activities that occur during normal development, such as maintaining a selective barrier for waste materials, inflammatory processes, coagulation, and other homeostatic functions [147].

**Table 3:** A short summary of different bioprinting techniques.

Material	3D Printing Method	Cells Used	Bioprinting Parameters	Ref.
Sodium alginate	Inkjet printing	3T3 mouse fibroblasts	Nozzle orifice diameter: 120 μm Voltage: 45 V Crosslinker: 2% CaCl_2_	[148]
Fibrin	Inkjet printing	Human microvascular endothelial cells	Droplet volume: 130 pL Post-printing: Incubated at 37 °C	[149]
Polycaprolactone+ Pluronic F127	Inkjet printing	Chondrocytes	Post printing: 0.25 MCaCl_2_	[150]
Alginate+ Gelatin	Inkjet printing	HL1 cardiac muscle cells	DC power supply: 4 V Crosslinker: CaCl_2_	[151]
Alginate+ Calcium deficient hydroxyapatite	Extrusion printing	Mouse osteoblasts	Nozzle diameter: 300 to 700 μm Crosslinker: 2.5% CaCl_2_	[152]
Oxidized methacrylated alginate	Extrusion printing	Human mesenchymal stem cells	Nozzle size: 20 G Cross linker: CaCl_2_	[153]
κ-Carrageenan/PVA	Extrusion printing	3T3 mouse fibroblasts	Physical crosslinking Printing speed: 2 to 7 mm/sec	[154]
Hyaluronic acid	Extrusion printing	3T3 mouse fibroblasts	Nozzle size: 25 G Extrusion flux: 0.33 mL/h Speed: 1.5 mm/sec	[155]
Silk fibroin	Digital light processing	3T3 mouse fibroblasts	Photoinitiator: LAP Digital micromirror device resolution: 30 μm Printing thickness: 50 μm	[100]
Glycidal methacrylate-hyaluronic acid+ Gelatin methacrylate	Digital light processing	Human induced pluripotent stem cells-hepatocytes	Photoinitiator: LAP UV Source: 365 nm	[156]
Multiwalled Carbon nanotubes	Digital light processing	NA	Photocurable resin: Acrylic resin- Are3d-dlp405	[157]
Poly (ethylene glycol) diacrylate+ Gelatin methacrylate	Stereolithography	3T3 mouse fibroblasts	Ultraviolet laser source	[158]
Gelatin methacrylate+ nanohydroxyapatite	Stereolithography	Fetal osteoblasts and human mesenchymal stem cells	UV laser source 200 μm diameter laser beam 5 mm/s printing speed.	[159]
Gelatin methacrylate+ graphene nanoplatelets	Stereolithography	Marrow-derived stem cells	UV laser source	[118]

LAP: Lithium phenyl-2,4,6-trimethylbenzoylphosphinate; NA: Not applicable.

### 4.3. Wearable Sensor Applications

Wearable sensor technology is of interest, especially for health monitoring as well as gesture control machines. Physical motion in the body leads to strain-sensing impulses, which leads to further conversion into electrical signals for effective sensing. For instance, Cui et al. demonstrated okara cellulose-based hydrogels that act as an effective transducer for motion sensing [160].

The doping of conductive ions with cellulosic fiber is an important area of electrical conductance for motion and transducer detection. Acrylic acid crosslinked allyl cellulose with the addition of urea, sodium hydroxide, and ammonium persulfate has been tested successfully and performed well even after 1000 cycles with varying electrical inputs [161].

### 4.4. Hydrogels for Humidity Sensors

**Figure 14:**
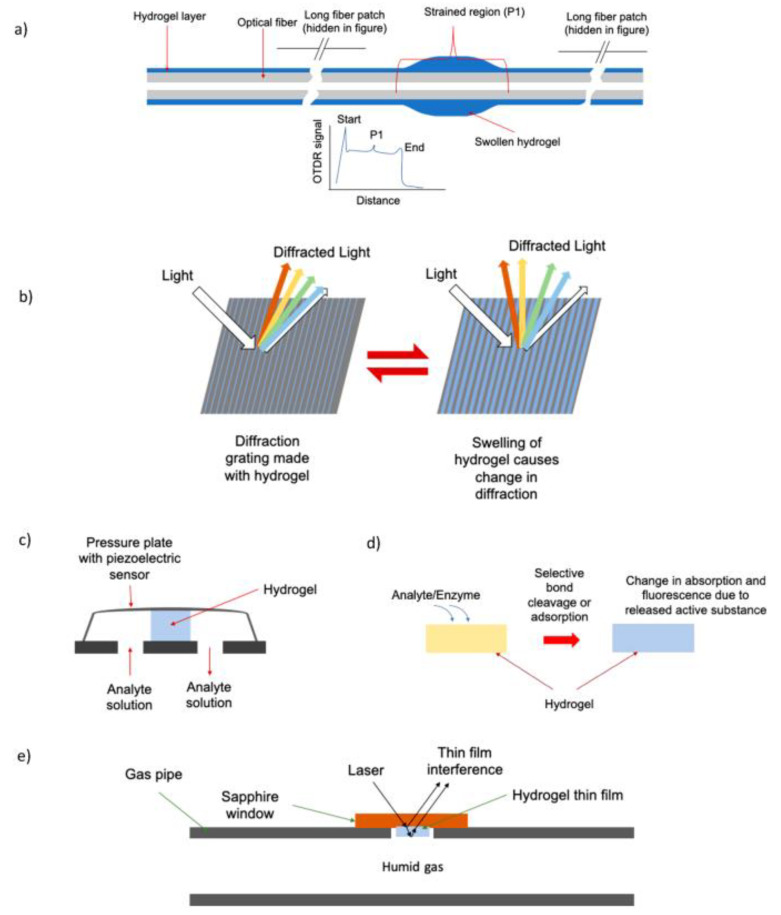
Schematic representation of the sensing mechanism using hydrogels [164]. (**a**) pH and ionic sensing, (**b**) fiber strain sensing by OTDR due to hydrogel swelling, (**c**) gas sensing: dissolved gas (acidic/basic) leads to hydrogel swelling followed by changes in the electric current [165]. (**d**) Molecular sensing: selective reactions lead to changes in fluorescence intensity due to the release of fluorophores on nanostructured materials. (**e**) Humidity sensor: changes in interference in the coating due to variation in thickness, which controls the swelling [163]. Copyright received.

A variety of hydrogels have been used as humidity sensors, wherein, the hydrogels are used to swell the sensor, resulting in changes in reflectance, optical path length, or easy ionic transportation, which improves the detection accuracy. The swelling behavior of the hydrogel leads to changes in the signal, which is subsequently reflected in the humidity-sensing recorder [162]. The changes in moisture in the surrounding environment absorbed by the hydrogel coating affect the refractive index, which leads to changes in the wavelength shift; therefore, humidity can be quantified with a relative precision of ±2.3% [162]. Furthermore, the accuracy of humidity sensing is dependent on the thickness of the coating. Buchberger et al. deposited poly (hydroxylmethyl methacrylate) on a sapphire substrate using the chemical vapor deposition (CVD) method with various film thicknesses to determine the variation in the relative humidity (RH) value with varying thickness (Figure 14). From their observations, they stated that changes in film thickness and optical light reflectance play a key role in determining RH values [163].

### 4.5. 3D Hydrogels for Water Purification

Some toxic chemical compounds lead to water pollution, thereby causing adverse effects not only on living things but also on the biological community [166]. Heavy metals present in their ionic forms are one of the major contaminants in water. According to Srivastava et al., more than 40 elements are considered heavy metals in water. Because of their persistence and toxic properties, heavy metals are receiving considerable attention among scientists, community activists, and government sectors. To remove water contaminants, various steps have been taken by researchers to protect the environment [167]. After a sufficient literature survey, we found that the adsorption method has received more extensive attention for effective water purification compared to physical and chemical methods.

To improve water purification efficiency, 3D hydrogels are used to remove heavy metal elements from contaminated water as well as other purification processes. Carboxymethyl cellulose (CMC) is one of the most common polymers used in water purification systems. Yang et al. successfully synthesized hydrogel beads by an inversion crosslinking method using an ECH crosslinking system [168]. The absorption capabilities of the hydrogels were tested with heavy metal ions such as Pb^2+^, Cu^2+,^ and Ni^2+^. The results revealed that the absorption efficiency was dependent on the formation of coordination bonds with oxygen in its carboxyl group. The formed hydrogel beads exhibited higher absorption efficiency of Pb^2+^, Cu^2+,^ and Ni^2+^.

Chitosan-based hydrogels (CSs) are tremendously used in environmental biotechnology due to their high absorption rate of heavy metals as well as cost-effectiveness. CS hydrogel beads are effectively used for heavy metal removal in industrial wastewater treatment [169]. Zhao and Mitomo blended CS with high concentrations of CMC (carboxy methyl cellulose) and synthesized a novel chitosan-based hydrogel called CMC-CS using irradiation-based crosslinking systems [170]. From the investigation, they stated that the adsorption capacity was improved by blending CS into the CMC system. Additionally, CS in addition to PVA showed strong adsorption behavior against lead [171]. Interestingly, they observed that effective lead absorption was strongly based on pH conditions and that the adsorption mechanism was based on ion exchange, complexation, and electrostatic interactions. During the electrostatic condition, the hydrogels follow the ȝ-potential.

### 4.6. Hydrogels for Smart Packaging Systems

The choice of material in the food packaging sector has always been a great concern. Food packaging can limit the loss of nutrition and flavor and extend the durability of food. Olden packaging systems using polymers are quite controversial due to their low degradability and toxicity to the environment [172]. Modern 3D hydrogel-based food packaging shows prominent results in its antimicrobial property and moisture barrier properties. Hydrogel-based materials used for food packaging systems exhibit several intrinsic characteristics such as (i) decreased water interaction with food and avoidance of food spoilage and bacterial invasion; (ii) prevention of moisture from dry fruits; (iii) adequate mechanical strength to maintain the structural integrity of the food; (iv) cost-effectiveness and ease of production; and (v) biodegradability to minimize environmental pollution [173].

Chitosan (CH) possesses excellent antioxidant and antimicrobial properties; thus, CH films have made significant contributions to food packaging industries. CH-based hydrogels are commonly used to preserve meat, fruits, and vegetables [174]. Roy et al. prepared PVP-blended carboxymethyl chitosan (CMCH) hydrogels and used them as packaging material for vegetables and fruits [175]. El-Mekawy et al. synthesized chemically crosslinked CH-based hydrogel films and achieved noteworthy properties such as flexibility, biodegradability, and softness for preserving meat [176]. From the investigation, they stated that CH-based hydrogels inhibited meat oxidation until 7 days and improved the shelf life of the meat. In addition, several researchers have found that CMCH-based hydrogels exhibited potent antimicrobial activity against *Staphylococcus* sp., *Pseudomonas* sp., molds, lactic acid, and yeasts, and were used in packaged raw beef as well as significantly increasing the shelf life of the food. Bandyopadhyay et al. developed a bacterial cellulose and guar gum-incorporated PVP-CMCH hydrogel film for blueberry packaging. From their investigation, they observed that the hydrogels kept blueberries fresh for 15 days. Moreover, the developed hydrogel film exhibited good degradability and showed 80% degradability within 28 days [177]. Based on the detailed investigation, CH-based hydrogels are a promising candidate for intelligent packaging systems.

## 5. Conclusions and Future Directions

Modern 3D hydrogels are promising materials for a variety of applications in numerous fields, including biosensors, drug delivery, and the food packaging sector, due to their embedding ability, stimuli response, water retaining property, biocompatibility, and nontoxicity. Bioprinting with 3D hydrogels has the virtue of high resolution of the input cells. Significant potential exists for employing this method in developing bioprinting technologies to print blood vessels, hearts, bones, cartilage, kidneys, skin, nerves, and other tissues. Molecules such as DNA can also be successfully printed, enabling research into the development and therapy of cancer. CH-based hydrogels are promising materials for food packaging systems. However, some challenges are recognized that should be addressed. There is still a need for further research and development on hydrogel material precursors, crosslinking and hydrogel-forming mechanisms, printability, printing parameters, features (mechanical, physics, biological, chemical), functionality, and applications—all for optimum 3D printing. Stimuli-based (pH and temperature) hydrogels in biosensor applications are relatively scarce, wherein creating a composite indicator is highly challenging (ripe/freshness indicator) and this needs to be focused on for better efficiency. Additionally, other indicators, such as microorganism indicators, leak indicators, and gas indicators using hydrogels, are quite limited. Therefore, the development of materials using hydrogels in these aspects is inevitable. Food packaging with QR codes or barcodes, which comprises the history of food, is of great interest. Hydrogels play an extensive role in improving the shelf life of food. The use of smart food packaging using hydrogels is still in the experimental stage and requires appropriate raw materials, repeated use rates, sensitivity, etc., for industrialization. Hydrogel-based 3D-printed tablets are used in heavy metal remediation. In particular, 3D-printed tripolymers, gelatin, sodium alginate, and PEI provide excellent activity in capturing heavy metals such as nickel, cadmium, cobalt, and lead.

The large-scale production of 3D-printed hydrogels in the holistic approach of bioremediation is quite challenging in the present scenario and needs further improvement. From the overall investigation, the present review concludes that a novel and easy hydrogel approach could help to fabricate inexpensive systems for wide applications.

## Figures and Tables

**Table 1:** A summary of 3D-printed conductive hydrogels for biomedical applications.

Material	Printing Method	Conductive Phase	Modification	Conductivity	Biomedical Application	Hydrogel	Biocompatibility	Ref.
Chitosan^MA^, Graphene^NS^	Bioplotting	Graphene 3 wt%	Addition of particle	0.0025	Tissue Engineering	Yes	Yes	[57]
GelMA, DNA-coated MWCNT	Bioplotting	CNT	Addition of particle	24 ± 1.8	Flex, Biosensors, Tissue Engineering	Yes	Yes	[58]
PEGDA, MWCNT-NH_2_	SL-Bioprinting	CNT	Addition of particle	2.21 ± 0.121 mC·cm^−2^	Tissue Engineering, Spinal cord repair	Yes	Yes	[59]
Cellulose nanofibrils, CNT	Bioplotting	CNT	Addition of particle	0.1–0.01, wet vs. dry	Biosensors	Yes	-	[60]
PANI, Phytic acid	Inkjet	PANI	In situ polymerization	0.23	Biosensors, medical electrodes	Yes	-	[61]
PNIPAM-L-CNT	Bioplotting	CNT	Addition of particle	0.016–0.02	Wearable Bioelectronics	Yes	-	[62]

## Data Availability

Not applicable.
